# Dr. David Little

**Published:** 1912-04

**Authors:** 


Dr. David Little, one of the oldest and most skillful of Rochester's physicians, died at his home, 162 Plymouth avenue, shortly before 3 p. m. March 1, 1912, aged 78 years. About two weeks before he was taken ill, and although he received all possible attention, his constitution, worn by years of hard and active service in the care of the sick and the advancement of his profession, was unable to throw off this illness.

David Little was born in Cherry Valley, Otsego county, February 9, 1834, the son of David and Julia Seelye Little. There he was educated at the academy, and from there he went to Union college, where he was a member of Kappa Alpha fraternity and Phi Beta Kappa society, being graduated in the class of 1855.

After graduating from Union college, Dr. Little attended the College of Physicians and Surgeons in New York city, being graduated in the class of 1857. He then served as interne at Bellevue hospital.

In 1859 he began the practice of medicine in Rochester, but at the outbreak of the Civil war he enlisted and was attached to the medical corps of the 13th infantry, New York volunteers, the regiment which saw so much fighting and was in the heat of many of the greatest battles of the war. For two years he served in this regiment, when he received an honorable discharge, and returned to Rochester once more to practice his chosen profession.

In 1863 he was married to Catherine A. Livingston, whom, with one daughter, Alice, and three sons, Dr. Seelye W., Beekman C. and Frank Little, he leaves.

Besides actively practicing medicine, Dr. Little spent considerable of his time in research and experiments, gaining for himself the distinction of being one of the closest students of the science, and one of the best and most skillful surgeons in the city.

After his return to Rochester he became surgeon and consulting surgeon on the staff of the City (now General Hospital, in which capacity he served nearly 50 years. His death comes as a distinct loss to the medical profession, and will be deeply felt; but not to the members of the profession alone, for his was a disposition of kindness for fellow man, and wherever he ministered to the wants of the sick he made a friend. Always looking for the comfort of others, his work was so kind that he might be called one of the truly great men of this generation.

Dr. Little was a member of Rochester Pathological Society, Hospital Medical Society, Monroe County Medical Society, Medical Society of the State of New York, and George H. Thomas Post, 4, Grand Army of the Republic (*Rochester Post Express*).

